# Analysis of intercondylar notch size and shape in patients with cyclops syndrome after anterior cruciate ligament reconstruction

**DOI:** 10.1186/s13018-021-02706-w

**Published:** 2021-09-08

**Authors:** Krzysztof Ficek, Jolanta Rajca, Jerzy Cholewiński, Agnieszka Racut, Paweł Gwiazdoń, Krzysztof Przednowek, Grzegorz Hajduk

**Affiliations:** 1 Department of Physiotherapy, The Jerzy Kukuczka Academy of Physical Education, 40-065 Katowice, Poland; 2 Deparment of Science, Innovation and Development, Galen-Orthopaedics, 43-150 Bieruń, Poland; 3 Department of Orthopedics and Traumatology, Brothers Hospitallers Hospital, 40-211 Katowice, Poland; 4 Department of Rehabilitation, Faculty of Health Sciences in Katowice, Medical University of Silesia in Katowice, Katowice, Poland; 5 Department of Biopharmacy, School of Pharmacy with the Division of Laboratory Medicine in Sosnowiec, Medical University of Silesia, 40-055 Katowice, Poland; 6 College of Medical Sciences, Institute of Physical Culture Studies, University of Rzeszow, 35-959 Rzeszow, Poland

**Keywords:** Cyclops lesion, Anterior cruciate ligament reconstruction, Intercondylar notch

## Abstract

**Background:**

Cyclops lesion is the second most common cause of extension loss after anterior cruciate ligament reconstruction. This study focused on the correlation between the anatomy of the intercondylar notch and the incidence of cyclops lesion. To determine whether the size and shape of the intercondylar notch are related to cyclops lesion formation following anterior cruciate ligament reconstruction according to magnetic resonance imaging (MRI) findings.

**Methods:**

One hundred twenty-five (125) patients were retrospectively evaluated. The notch width index (NWI) and notch shape index (NSI) were measured based on coronal and axial MRI sections in patients diagnosed with cyclops syndrome (*n* = 25), diagnosed with complete anterior cruciate ligament (ACL) tears (*n* = 50), and without cyclops lesions or ACL ruptures (*n* = 50).

**Results:**

Imaging analysis results showed that the cyclops and ACL groups had lower mean NWI and NSI values than the control group. Significant between-group differences were found in NSI (*p* = 0.0140) based on coronal cross-sections and in NWI (*p* = 0.0026) and NSI (*p* < 0.0001) based on axial sections.

**Conclusions:**

The geometry of the intercondylar notch was found to be associated with the risk of cyclops lesion formation and ACL rupture.

## Background

Cyclops lesion, defined as the local presentation of arthrofibrosis, is the second most common cause of extension loss after anterior cruciate ligament (ACL) reconstruction [1]. In the early postoperative period, cyclops syndrome, described by Jackson and Schaefer [2] in 1990, causes extension loss of approximately 5° compared with a healthy lower limb. A cyclops lesion is a fibrous nodule of granulation tissue anterolateral to the tibial tunnel that has matured in a manner similar to a healing scar and occasionally develops cartilaginous or bony tissue, and it is usually not associated with any clinical symptoms of the knee [2, 3]. The incidence of cyclops syndrome in patients after ACL reconstruction ranges from 1.9 to 10.6%, whereas the incidence of cyclops lesions that do not cause extension loss ranges from 2.2 to 46.8% [4–11]. Most of these reports are based on single-bundle ACL reconstruction.

Several risk factors have been linked to loss of knee motion, including the mechanism of ACL injury and associated injuries, the timing of surgery, technical factors, and postoperative/rehabilitation factors [12]. Among the factors associated with surgery, the timing of surgery remains particularly controversial. Many authors have reported an association between early surgery and the development of arthrofibrosis [13–16], while others have found no relationship between the timing of surgery and extension loss [17–21]. Patients whose ligaments were reconstructed within the first week of injury had a statistically significant increase in the incidence of motion loss compared with those who waited at least 3 weeks [15]. Wasilewski et al. [22] showed that acute ACL reconstruction (5–10 days after injury) significantly slowed down postoperative motion recovery compared to delayed surgery.

On the other hand, not only the timing of the surgery but also the condition of the knee prior to surgery may be important. The goals of preoperative rehabilitation are to restore normal knee range of motion, eliminate swelling, and regain leg control [15]. Preoperative motion is an important preoperative predictor of ultimate motion and may even be the key clinical factor to guide decisions regarding the timing of ACL reconstruction [23].

As genetic factors play a proven role in ACL rupture [24, 25], several genetic mechanisms have been proposed to be related to arthrofibrosis after ACL reconstruction. Platelet-derived growth factor-β (PDGF-β) and transforming growth factor-β (TGF-β) may play a central role in the healing process, but their expression has been associated with unresolved inflammation and fibrotic events [26, 27].

Another risk factor that may be related to the formation of a cyclops lesion is associated with the anatomy of the femoral intercondylar notch. The geometry of the intercondylar notch varies among the population, including the differences between females and males. Three geometry types of intercondylar notch have been distinguished: (1) A-shaped, (2) U-shaped, and (3) W-shaped [28]. The A-shaped notch is defined as a stenotic notch which is narrow from the base to the midsection as well as at the apex. In the U-shaped notch, the midsection does not narrow, allowing for a wider contour to the notch. The W-shaped notch is similar to type U, but with two apparent apices [29]. Interestingly, Hirtler et al. [30] performed a study in which they described the ongoing dynamic morphologic modifications in the intercondylar notch at different stages of life, indicating that the shape and size of the notch and of the femoral condyles changed significantly during life. Many authors have reported that narrowing of this notch is related to an increased risk of ACL injury [31–36]. Based on a recent study, it can be predicted that a smaller notch might be correlated with ACL impingement [37]. Moreover, a cause of notch impingement could potentially exist for a “mismatch” between the notch size and the ACL size [38]. However, the literature seldom mentions the association between the notch size and the formation of cyclops lesions. Some authors reported that notch size influences cyclops formation [39], and some did not find statistically significant differences in the size of intercondylar notches in the patient groups with and without cyclops lesions [40].

Therefore, the purpose of this study was to retrospectively determine whether the size and shape of the intercondylar notch were related to cyclops lesion formation. The authors predict that the geometry of the intercondylar notch might be a risk for developing cyclops lesions.

## Methods

### Patients

**Fig. 1:**
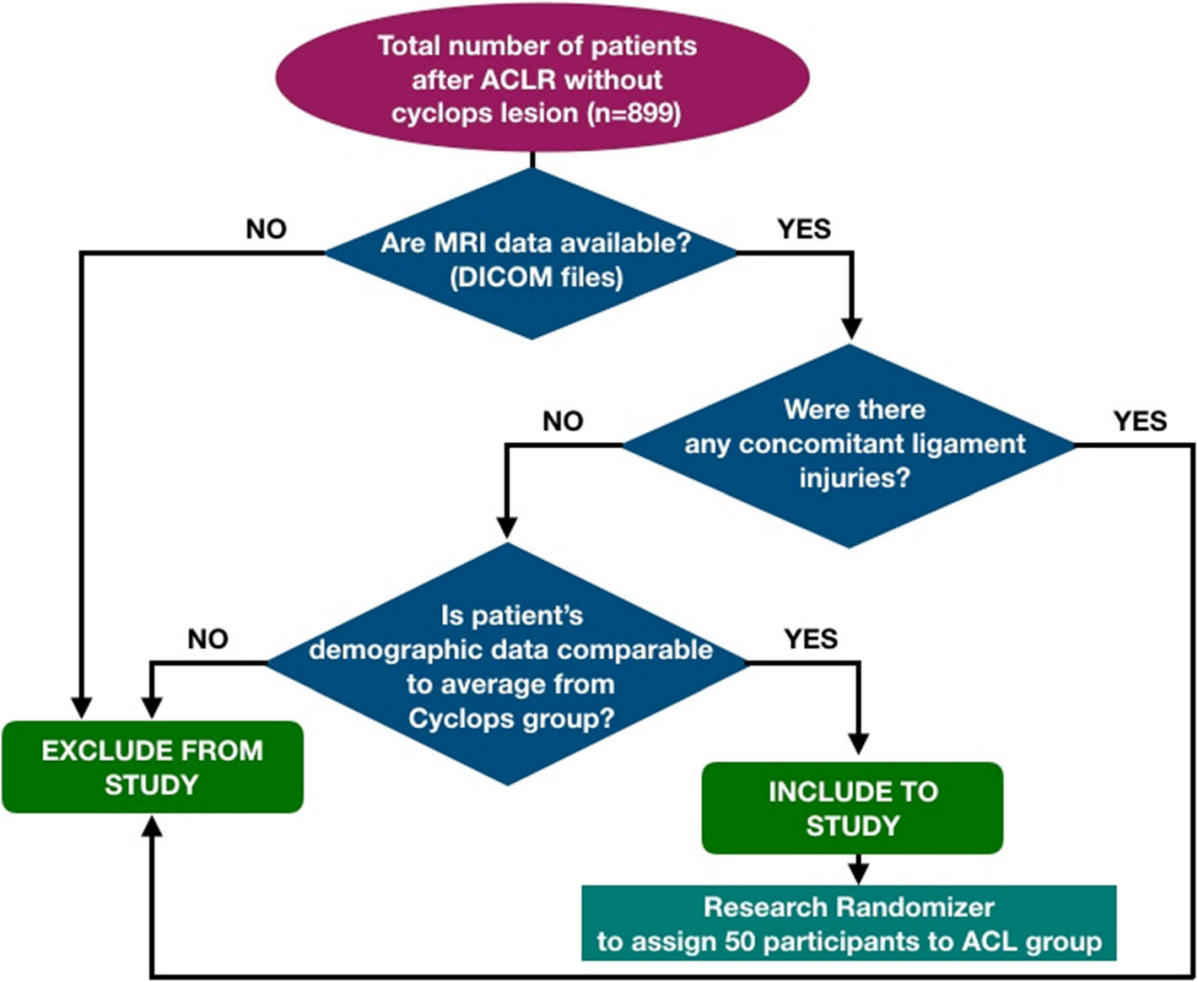
Patients selection algorithm. The question about demographic data refers to age, sex, and BMI

There were 929 patients who underwent surgery due to complete ACL tears between 2011 and 2017, and we reviewed the medical records of the patients treated at our clinic for cyclops syndrome. We identified thirty (30) patients diagnosed with cyclops lesions after ACL reconstruction. All these cases were confirmed by MRI scans and arthroscopic surgery, during which the lesion was removed. The major criterion for exclusion was the lack of digitally available MRI data on the knee joint. Therefore, the cyclops group included 25 patients. Among the remaining patients with available MRI data, we randomly selected 50 patients to be included in the ACL group, and they had demographic characteristics similar to those of the cyclops group. The algorithm for the patients’ inclusion and exclusion is presented in Fig. 1. The control group (*n* = 50) included patients without rupture of the ACL or other ligaments in the knee who underwent MRI scans of the knee due to suspicion of a meniscus tear and did not have any reports of knee instability in the medical records. No patient in the selected cohort underwent notchplasty during ACL reconstruction. The demographic data are displayed in Table 1.

**Table 1:** Demographic data

	Cyclops (***n*** = 25)	ACL (***n*** = 50)	Control (***n*** = 50)	p*	Cyclops-ACL	Cyclops Control	ACL-Control	Cyclops+ACL-Control
				Chi-squared test				
**Sex (male:female)**	17:9	37:13	35:15	0.8385	0.5878	0.8594	0.6560	0.8848
**Knee (left:right)**	18:7	25:25	27:23	0.1716	0.0655	0.1336	0.3889	0.8537
				K-W	Bonferroni test			
**Age (years)**	32.7 ± 8.8	32.7 ± 10.1	38.0 ± 13.7	0.0072*	0.9999	0.0151*	0.0106*	0.0009*
**Body weight**	75.7 ± 14.4	80.2 ± 18.6	82.7 ± 19.4	0.3994	0.5378	0.3850	0.9999	0.0241*
**Body height**	177.6 ± 9.8	176.6 ± 8.4	173.5 ± 18.2	0.7016	0.7741	0.8734	0.9999	0.4368
**BMI**	23.8 ± 2.9	25.5 ± 4.8	26.0 ± 4.8	0.1540	0.1969	0.0855	0.9071	0.1140
**Graft diameter**	0.77 ± 0.10	0.79 ± 0.05	–	–	0.4266	–	–	–

*K-W* Kruskal-Wallis test

*Statistically significant (*p* < 0.05)

### Surgical procedure and postoperative management

The transportal femoral tunnel drilling technique for endoscopic ACL reconstruction was used in patients in the cyclops and ACL groups. The types of grafts used during the procedure are presented in Table 2. In patients with additional features of *I*° medial instability, the quadriceps tendon or the patellar tendon was used.

**Table 2:** Types of grafts used in ACL reconstruction in the cyclops and ACL groups

	Cyclops [%]	ACL [%]
**ST GR**	83	69
**Quadriceps tendon**	13	12
**Patellar ligament**	4	–
**Rectus femoris tendon**	–	2
**Allogeneic**	–	16

After they were harvested, the semitendinosus and the gracilis tendon (ST GR) were doubled and folded over the loop of an EndoButton CL (Smith & Nephew Inc., Andover, MA, USA), and the distal ends of the grafts were sutured. When the quadriceps tendon was harvested, the operator sutured the tendon on both ends with Krakow sutures. Next, the diameter of each graft was measured using a cylindrical gauge (sizing system, Acufex, Smith & Nephew Inc., Andover, MA, USA). All grafts were pretensioned in full extension, with 20 lbs applied by a tensiometer (Smith & Nephew Inc., Andover, MA, USA). The surgeon removed only the interposed tissue of the ACL remnant. The knee was flexed to 120°, and a guided pin was placed in the anatomical femoral footprint using a femoral guide. Then, the guided pin was overdrilled using a 4.5 drill and then a 7–9-mm-diameter cannulated reamer, and the diameter depended on the diameter of the whole graft. A 5-mm unreamed bone fragment was left to anchor the EndoButton plate. Next, the knee was flexed to 90°, and a guided pin was placed using a tibial guide. A tibial tunnel was created. Femoral fixation was achieved using an EndoButton system (Smith & Nephew Inc., Andover, MA, USA) or with an interference screw Biosure PEEK system (Smith & Nephew Inc., Andover, MA, USA), depending on the graft used. Tibial fixation was performed with a Biosure PEEK interference screw. Repeatedly in the last phase of the surgical procedure, the operator checks for any impingement with the intercondylar notch by flexing and extending the knee. If necessary, the adequate debridement of damaged parts of ACL remnant was performed to avoid creating a potential fibrous conflict, simultaneously trying to keep as much remnant tissues as possible, due to the fact that remnant preservation enhances early revascularization of the graft [41].

All patients started rehabilitation protocols 1 week after surgery. A standardized physical therapy program was prescribed for all patients. Cyclops nodules were diagnosed by the presence of pain, loss of motion (extension and/or flexion), and audible clicking during the terminal phase of extension and were confirmed by MRI.

### MRI-based measurement of the femoral intercondylar notch size and shape

**Fig. 2:**
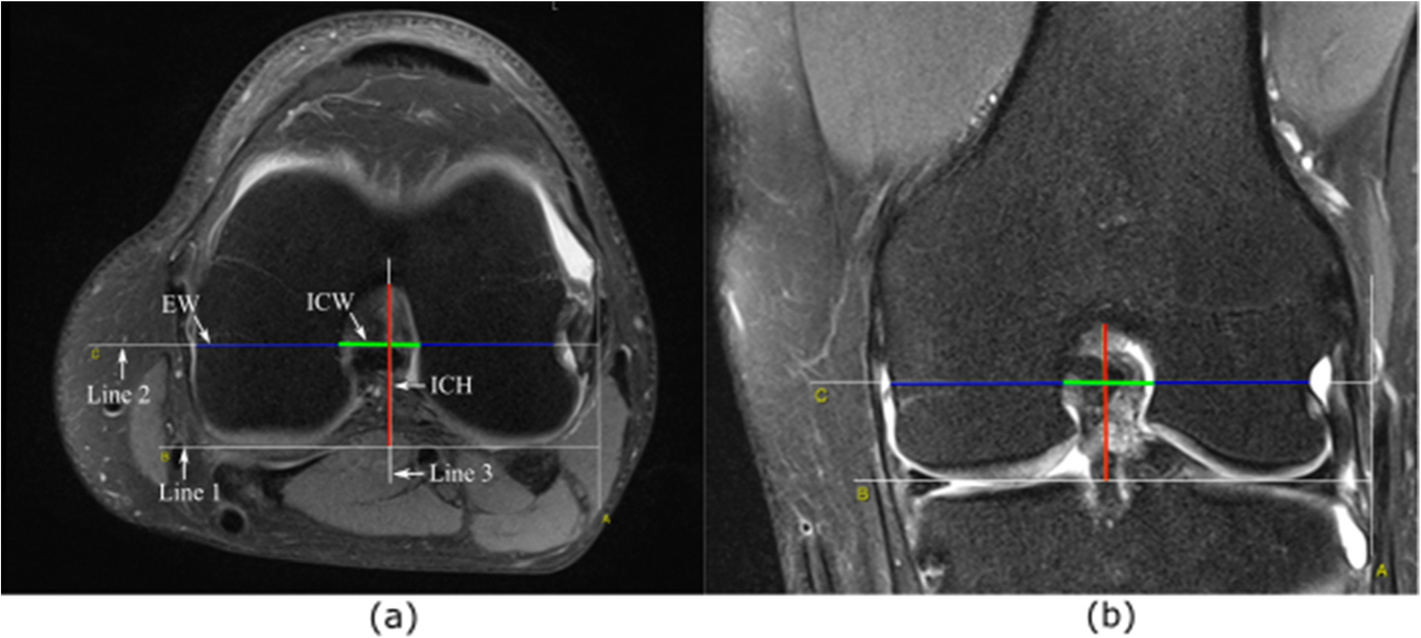
Measurement of the intercondylar notch. On axial cross-section (**a**). On coronal cross-section (**b**)

Two independent examiners performed each MRI measurement, and the average of these measurements was recorded and used for further analysis. An open-source platform, OsiriX MD (v. 7.5, Osiris Foundation, Geneva, Switzerland), was used for image analysis. The MRI images were analyzed in a random order. The MRI measurements were performed on the coronal T2 scans and axial T2 scans. The measurement method was based on the approach proposed by Fuji et al. [39]. We confirmed Blumensaat’s line on a T2-weighted sagittal plane beside the lateral intercondylar wall, and then, we identified the coronal and axial planes of the middle point of Blumensaat’s line. On the scans, the full contours of the medial and lateral condyles, the notch shape, and a groove of the popliteus tendon sulcus of the lateral condyle were clearly visible. On these scans, three lines were designated (Fig. 2). Line 1 was designated as the line between the lowest points of the cartilage surfaces of the medial and lateral condyles. A line parallel to line 1 and passing through the largest groove of the popliteus tendon sulcus was defined as line 2. With respect to line 2, the intercondylar notch width (ICW) was measured. The distance measured on line 2, determined by the points of intersection of the line with the lateral and medial walls of the condyles, was marked as the epicondyle width (EW). The height of the intercondylar notch was measured on line 3, which was perpendicular to line 1. The intercondylar notch height (ICH) was defined as the distance from the level of the femoral joint surface (line 1) to the top of the notch. The ratio of ICW to EW represented the notch width index (NWI), as shown in Eq. (1), and that of ICW to ICH represented the notch shape index (NSI), as shown in Eq. (2).

1 $$ \mathrm{NWI}=\frac{\mathrm{ICW}\ \left[{\mathrm{cm}}^2\right]}{\mathrm{EW}\left[{\mathrm{cm}}^2\right]} $$

2 $$ \mathrm{NSI}=\frac{\mathrm{ICW}\ \left[\mathrm{cm}\right]}{\mathrm{ICH}\left[\mathrm{cm}\right]} $$

The femoral intercondylar notch sizes and graft sizes were compared between the knees with cyclops lesion (cyclops group) and those without cyclops lesions (ACL group). In order to determine the effect of the size mismatch between the graft and the intercondylar notch on cyclops lesion formation, the ratio between the graft diameter and intercondylar notch width (G/ICW) was calculated and compared between the two groups.

### Statistical analysis

The statistical analysis was performed using the Statistica software (release 10.0, StatSoft, Tulsa, OK, USA) and GNU R software with an additional package to compare the differences among the tested groups. The normality (Shapiro-Wilk test) and the homogeneity of variance (Levene’s test) of all the measured variables were checked. A nonparametric test (Kruskal-Wallis test) was used to assess the differences among the cyclops, ACL, and control groups. For multiple comparisons, the Bonferroni test was used. The chi-square test was used to compare the qualitative data (sex and knee) between the groups. The G/ICW results were statistically analyzed by the Mann-Whitney *U* test between the cyclops and ACL groups. For all tests, differences with *p* < 0.05 were regarded as significant. Interobserver reliability between two observers (for parameters ICW, ICH, and EW, both for coronal and axial cross-sections) was evaluated using the intraclass correlation coefficient (ICC). As a general guideline [42], ICCs exceeding 0.75 are indicative of good reliability, whereas those below 0.75 indicate poor to moderate reliability.

### Ethics

The study was performed according to the Declaration of Helsinki. The study protocol was approved by the Research Ethics Committee of the Jerzy Kukuczka Academy of Physical Education (Reference Number: 2/2011). Due to the retrospective nature of the study, the requirement for informed consent was waived.

## Results

**Fig. 3:**
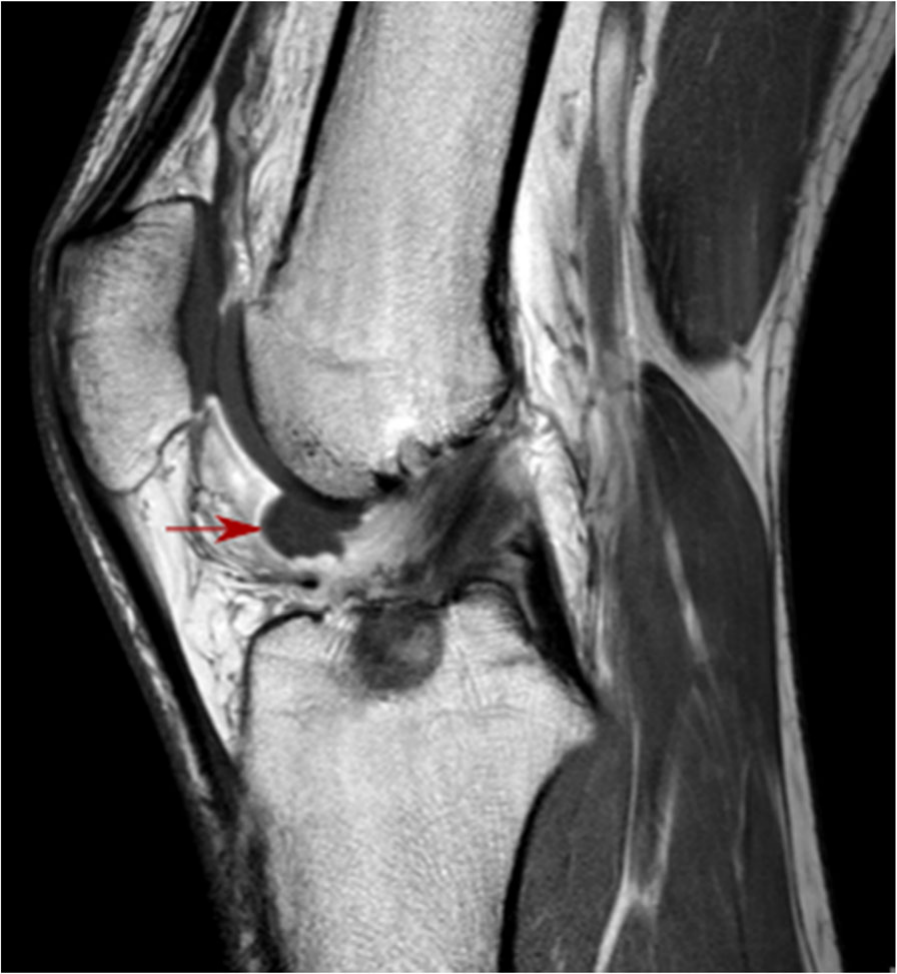
Cyclops lesion

The MRI images of the cyclops group, which were reviewed by a musculoskeletal radiologist, showed an abnormal signal anteriorly to the ACL graft in the intercondylar notch (Fig. 3). The cyclops lesion was detected on the arthroscopy look when the knee was extended. Patients received arthroscopic surgery for the removal of the lesion, and in every patient, the loss of extension in the knee joint was improved, followed by rehabilitation protocol, equal for all patients. However, in six patients (from the cyclops group (*n* = 25)), symptomatic cyclops lesion reappeared in the 6 to 24 months after. In the subsequent control, MR imaging as well as during the consecutive arthroscopic surgery with lesion removal, the variability in increment of the diameter and volume of the graft which underwent ligamentization was observed. The notchplasty was performed during the second arthroscopy with debridement of excess fibrous tissue, which formed from uncontrolled scarring response, surrounding the ACL graft. Therefore, the second-look arthroscopy comprised invasive widening of the femoral notch to avoid another return of cyclops lesion.

**Fig. 4:**
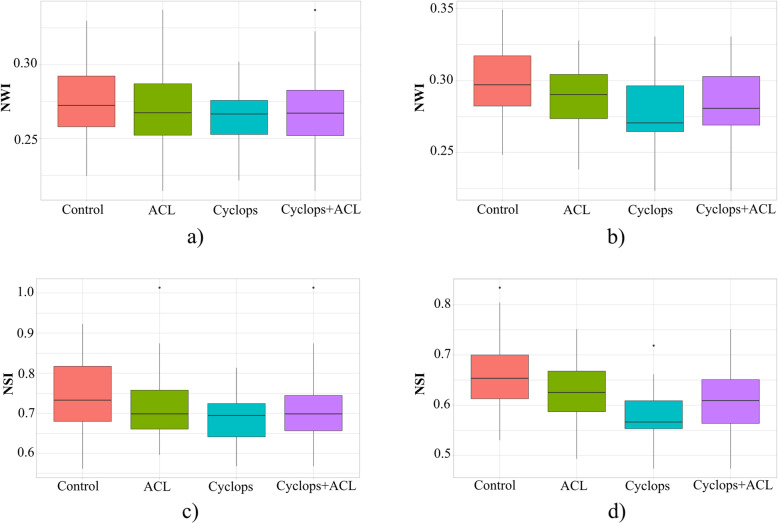
Boxplots showing the NWI and NSI results based on coronal cross-sections (**a**, **c**) and axial cross-sections (**b**, **d**). Boxplot legend: line inside = median, box = 25th and 75th percentiles, whiskers = maximal and minimal value

The comparison of the NWI and NSI between the tested groups is shown in Table 3. Compared with the control group, the cyclops and ACL groups demonstrated a lower mean NWI and NSI. The results of the statistical analysis are presented in Fig. 4 and Table 4. Significant differences were found in the coronal cross-sections between the cyclops and control groups in NSI (*p* = 0.0086), in the axial cross-sections between the cyclops and control groups (*p* = 0.0016) and between the ACL and control groups (*p* = 0.0284) in the NWI, and between the cyclops and ACL (*p* = 0.0037), cyclops and control (*p* = 0.0001), and ACL and control groups (*p* = 0.0339) in the NSI. The analysis between the patients who underwent reconstructive surgery (Cyclops+ACL) and the control group shows that there are significant differences in the coronal cross-sections in NSI (*p* = 0.0035) and in the axial cross-sections in both parameters NWI (*p* = 0.0007) and NSI (*p* = 0.0001).

**Table 3:** Comparison of NWI and NSI between the groups

		Mean	SD	Median	95% confidence interval	
Cross-section: coronal						
NWI	Cyclops	0.2629	0.2665	0.2665	0.2541	0.2717
	ACL	0.2689	0.0247	0.2672	0.2619	0.2759
	Cyclops+ACL	0.2669	0.0236	0.2671	0.2615	0.2723
	Control	0.2767	0.0255	0.2723	0.2695	0.2840
NSI	Cyclops	0.6871	0.0629	0.6940	0.6611	0.7130
	ACL	0.7148	0.0793	0.6982	0.6922	0.7373
	Cyclops+ACL	0.7056	0.0750	0.6982	0.6882	0.7228
	Control	0.7456	0.0878	0.7326	0.7207	0.7706
Cross-section: axial						
NWI	Cyclops	0.2790	0.0263	0.2703	0.2681	0.2898
	ACL	0.2863	0.0232	0.2381	0.2797	0.2929
	Cyclops+ACL	0.2838	0.0243	0.2805	0.2782	0.2894
	Control	0.2986	0.0229	0.2970	0.2921	0.3051
NSI	Cyclops	0.5800	0.0561	0.5664	0.5569	0.6032
	ACL	0.6285	0.0600	0.6252	0.6114	0.6455
	Cyclops+ACL	0.6123	0.0627	0.6088	0.5979	0.6268
	Control	0.6643	0.0229	0.6534	0.6440	0.6846

**Table 4:** Results of Kruskal-Wallis analysis

	Kruskal-Wallis	Cyclops-ACL	Cyclops-Control	ACL-Control	Cyclops+ACL-Control
Coronal section					
NWI	0.1239	0.6335	0.0793	0.2475	0.0301*
NSI	0.0140*	0.3847	0.0086*	0.0690	0.0035*
Axial section					
NWI	0.0026*	0.2644	0.0016*	0.0284*	0.0007*
NSI	0.0000*	0.0037*	0.0001*	0.0339*	0.0001*

*Statistically significant (*p* < 0.05)

The ratio between the graft diameter and ICW (G/ICW) was comparable in the cyclops and ACL groups (Table 5).

**Table 5:** Results of G/ICW based on coronal and axial cross-sections

	Mean ± SD	Median	*p*
Cross-section: coronal			
Cyclops	0.4119 ± 0.0555	0.4020	0.6559
ACL	0.4038 ± 0.0446	0.4092	
Cross-section: axial			
Cyclops	0.3851 ± 0.0555	0.3920	0.5925
ACL	0.3718 ± 0.0689	0.3734	

Within the cyclops, ACL, and control groups, we did not find significant differences in NWI and NSI between male and female patients.

For the mean interrater reliability between observers 1 and 2 for all component parameters (ICW, ICH, EW) of NWI and NSI, the ICC of the analyzed features in the coronal and axial cross-sections was 0.75 and 0.90, respectively. Considering the axial cross-sections, there was no parameter for which ICC was below 0.75. All ICCs showed statistical significance (*p* < 0.05). According to the guidelines of Portney and Watkins [42], reliability for many clinical measurements should exceed 0.90 to ensure reasonable validity. Thus, outcomes of NWI and NSI based on coronal cross-sections should be treated with some degree of caution.

## Discussion

In our study, we investigated the geometry of the intercondylar notch by measuring the width and shape index using MRI on the coronal and axial cross-sections to identify the associations between the geometry and the occurrence of cyclops syndrome. However, considering the moderate interrater reliability for coronal cross-sections between the two observers, conclusions were based on the results for axial cross-sections (mean ICC = 0.90). The most important finding of this research was that patients who developed cyclops lesions had significantly lower NSIs than patients with and without ACL rupture or other ligaments in the knee (ACLs and controls, respectively). According to our results, the shape of the femoral intercondylar notch, described by NSI, might be a potential risk factor for the development of cyclops lesions. In our study, we also used a previously known NWI parameter, which also confirmed our prediction about its influence on graft impingement against the notch. Therefore, our hypothesis regarding the association of the geometry of the femoral notch with an increased occurrence of cyclops syndrome was confirmed based on the axial cross-sections. A superior measure of notch geometry appears to be NSI, which is a relative measure of notch width in the medial/lateral direction to the notch height in the anterior/posterior direction. Knees with lower NSI may not permit normal function of the ACL [43]. According to Tillman et al. [43], when the knee is in full extension, the ACL is pulled tight and will reside in the more anterior portion of the intercondylar notch. Therefore, a low NSI indicates that this particular region of the intercondylar notch will be narrower and thus will provide less space for the ACL to function correctly.

According to previous studies [39, 40] and to the assumption that the width of the intercondylar notch is important in developing cyclops lesion, we calculated the ratio between graft diameter and notch width (ICW) to determine the potential size mismatch between the graft and intercondylar notch width (G/ICW). The results showed no significant differences between patients after ACL reconstruction with and without cyclops lesion; hence, not the width alone but the shape may have an impact on impingement of the graft. The literature reports that excessively large grafts may increase the risk of graft notch mismatch, impingement, loss of extension, and ultimately failure [44]. A great majority of studies indicate that intercondylar notch stenosis may be a cause of ACL rupture because of impingement of the ACL. It is suggested that any rotational force while the knee joint is near full extension increases the potential of ACL tear during knee valgus through shearing or impinging mechanisms [45].

The diagnosis of cyclops syndrome is usually based on clinical symptoms such as painful extension during walking, snapping, and progressive extension loss. Rehabilitation does not bring pain relief and cannot improve the extension loss in the developed phase of the syndrome. In suspicion of cyclops syndrome, MRI is necessary. The etiology of cyclops formation is multifactorial and has not yet been clearly explained; however, there are several hypotheses regarding it: cartilage and bone residue in the joint following tibial tunnel drilling and preparation for graft passage [2, 11], torn graft fibers [46], the native ACL stump [47], and repeated graft impingement on the notch [48]. Pinto et al. [47] note the important role of rehabilitation deficits manifested by hamstring contracture. According to Noailles et al. [49], factors that were not associated with the occurrence of cyclops lesions are age [47, 50], level of sports activities [47], occurrence of bone bruises [47], use of patellar or hamstring graft [4, 6, 47, 50], preservation of residual ACL fibers [10], concomitant injuries (meniscal or antero-lateral ligament reconstruction) [39, 47, 50], and time from surgery to the first rehabilitation session [6, 39].

Another hypothesis concerns narrow intercondylar notches. To date, the size of the intercondylar notch has mostly been associated with ACL rupture. Patients with narrow intercondylar notches are considered more susceptible to ACL injury. The results of studies [36, 51] have shown that NWI and NSI in patients with ACL rupture were significantly lower than those in the control group. Many studies have confirmed that the shape and width of the femoral intercondylar notch, calculated on coronal cross-sections in MRI, are important factors that affect ACL rupture [36]. In contrast, Teitz et al. [52] and Schickendantz and Weiker [53] found no significant correlation between notch dimensions and risk of ACL injury. In our study, we found significant correlations based on axial and coronal cross-sections, by comparing two groups cyclops and ACL (Cyclops+ACL) to the control group. On the other hand, the literature rarely draws attention to the relation between notch size and cyclops formation. Fujii et al. [39], based on bi-socket ACL reconstruction, concluded that patients with small intercondylar notches tend to develop cyclops lesions. There was a significant correlation between notch size and cyclops formation. They predicted that the formation of cyclops lesions may depend on notch impingement induced by the size mismatch between the intercondylar notch and the graft. The authors suggest performing measurements on MRI preoperatively to avoid mismatch of the size of the graft to the intercondylar notch [39]. According to these results, a narrow intercondylar notch might be a potential risk factor for developing cyclops lesions due to graft impingement against the notch. On the other hand, another study [40] showed no differences between cyclops formation and notch size. Both of these studies have limitations. In Fujii et al.’s [39] research, the transtibial technique was applied, which is not an anatomical method; therefore, it may affect the incidence of cyclops syndrome. In Bradley et al.’s [40] research, the nonanatomic transtibial technique was also used, and different measurement methods of the intercondylar notch were applied. In our study, we reported that the impingement was checked in each patient by flexing and extending the knee by the operator, and none of the patients from the cyclops and ACL group had impingement with intercondylar notch—none of these patients underwent notchplasty. However, in six patients, symptomatic cyclops lesion reappeared; therefore, the second arthroscopy comprised widening of the femoral notch to avoid a return of cyclops lesion. Since long-term consequences of notchplasty are questionable, and several studies reported detrimental effects such as destructive effect on the near cartilage, negative biomechanical effects on the graft, and postoperative bleeding caused by notchplasty that may lead to arthrofibrosis [54], it was decided that widening of the femoral intercondylar notch would not be performed during the first removal of cyclops lesion. Additionally, with regard to the unpredictable direction of ligamentization, deeper evaluation of the notch in preoperative planning for ACL surgery based on diagnostic imaging in patients with narrow notches offers the opportunity to tailor the size of ACL remnant which is used during the ACL reconstruction. The preservation of ACL remnant helps the biological process of graft healing, enhances early revascularization of the graft, and may improve recovery of joint positioning, which is crucial for the rehabilitation process [41]. However, it increases the volume of the graft. For that reason, detailed analysis of the intercondylar notch may facilitate the operator’s decision about reducing the volume of ACL remnant in case of narrow geometry of the notch, which, in the end, increases the likelihood of cyclops lesion formation.

In the literature, we did not find any papers in which both width and shape were evaluated in relation to the incidence of cyclops syndrome. To the best of our knowledge, no research has assessed the NSI in MRI in axial cross-sections. The majority of research papers related to intercondylar notches present MRI measurements performed only on coronal sections. In our paper, we decided to assess NWI and NSI parameters on the axial cross-sections to verify and compare the outcomes from two reconstructed sets of images. In fact, the results obtained from the axial section showed higher interrater reliability between two observers and accordingly higher statistical significance.

This study has some limitations. We did not perform microscopic analysis of the extracted nodules and were not able to determine their histologic formation. Another issue is that part of the control MRI was performed in an external center; therefore, the MRI examination protocols varied. Additionally, in order to draw a solid conclusion, it will be necessary to increase the sample size in the cyclops group (*n* = 25) because the ACL and control groups are twice as large (*n* = 50).

## Conclusions

In conclusion, our goal was to shed new light on the size analysis of the femoral intercondylar notch, which until now was mostly carried out in a group of patients diagnosed with ACL rupture. Traditional measures of NWI in patients with cyclops syndrome address the question about the influence of narrow notches on the risk of developing this type of lesion based on axial cross-sections. However, we propose MRI analysis, including both coronal and axial sections, not only focusing on NWI parameters but also quantitatively analyzing the shape of the femoral intercondylar notch, which is significantly different in control subjects. In our opinion, the NSI parameter may be of great importance in evaluating notch-graft conflict and thus can have an impact on the incidence of appearing cyclops lesion. However, additional studies should be conducted using larger samples for the results to be confirmed.

## Data Availability

The datasets will be available from the corresponding author on reasonable request.

## Abbreviations

ACL: Anterior cruciate ligament
PDGF-β: Platelet-derived growth factor-β
TGF-β: Transforming growth factor-β
ICW: Intercondylar notch width
EW: Epicondyle width
ICH: Intercondylar notch height
NWI: Notch width index
NSI: Notch shape index
